# Mechanisms influencing adult children’s willingness to use medical visit accompaniment services for older adults in Nanjing: a structural equation modeling study

**DOI:** 10.3389/fpubh.2026.1852438

**Published:** 2026-05-28

**Authors:** Shuxin Dong, Huanhuan Liu, Yiming Yao, Siying Wang, Jie Li, Junlong Shen

**Affiliations:** School of Health Economics and Management, Nanjing University of Chinese Medicine, Nanjing, China

**Keywords:** adult children of older adults, influencing factors, medical visit accompaniment services, structural equation modeling, theory of planned behavior, use intention

## Abstract

**Objective:**

In view of the current problems of insufficient service supply and low public awareness in the medical visit accompaniment service market, this study aimed to explore the key factors influencing adult children’s willingness to use medical visit accompaniment services for older adults, so as to provide a theoretical basis for optimizing medical supportive services and resource allocation.

**Methods:**

A questionnaire survey of 638 adult children of older adults in Nanjing was used as the study sample. Based on the Theory of Planned Behavior, latent variables including attitude toward behavior, subjective norm, and perceived behavioral control were incorporated to construct a cognitive path model of adult children’s decision-making in choosing medical visit accompaniment services. Structural equation modeling was conducted using Amos 28.0, followed by path analysis and effect decomposition, to examine how different types of needs influenced actual behavioral willingness.

**Results:**

Basic support needs positively influenced willingness to use medical visit accompaniment services through mediating pathways and represented the strongest driving factor. In contrast, emotional and dignity-related needs and health management needs showed negative effects because of insufficient service provision.

**Conclusion:**

Willingness to use medical visit accompaniment services is shaped by multiple factors. Policy and service planning should prioritize standardized basic support, gradually develop emotional and health-management-oriented service modules, and improve service accessibility, equity, and social recognition through multi-stakeholder collaboration.

## 1 Introduction

China’s population aging is accelerating rapidly. According to the 2024 Statistical Bulletin on the Development of China’s Aging Undertakings, by the end of 2024, people aged 60 years and above accounted for 22.0% of the total population, while those aged 65 years and above accounted for 15.6%. The degree of population aging in Nanjing has also been intensifying year by year. The 2023 Report on the Older Population Information and the Development of Aging Undertakings in Nanjing showed that by the end of 2023, among Nanjing’s resident population, 21.97% were aged 60 years and above and 16.01% were aged 65 years and above.

In 2024, the Opinions of the General Office of the State Council on Developing the Silver Economy and Enhancing the Well-being of Older Adults, as China’s first national-level policy document specifically targeting the silver economy, systematically proposed 26 concrete tasks for its development. The 14th Five-Year Plan for the Development of the National Aging Undertakings and Older Adult Care Service System further emphasized the need to increase the supply of health services for older adults, optimize resource allocation, expand the workforce, improve the health status of older adults and the accessibility of medical services, and promote the development of the smart health and older adult care industry (1, 2). Guided by these national strategies, the development of an age-friendly healthcare environment has become a crucial pillar supporting the silver economy (3, 4).

As an important medical hub in East China, Nanjing has a high concentration of quality healthcare resources. By the end of 2023, the city had 3,846 medical and health institutions, including 311 hospitals. Among them, 38 were tertiary Grade A hospitals, accounting for 35% of the total in Jiangsu Province, attracting not only local residents but also a large number of patients from other regions. However, behind this high-density distribution of medical resources, older patients often face multiple challenges in the actual process of seeking healthcare, including difficulties in registration, communication, and referral (5–8). Traditionally, these problems have been addressed through family accompaniment. In real life, however, an increasing number of middle-aged and younger adults are unable to provide routine accompaniment because of time constraints, geographical distance, and work-related pressure (9, 10). Against this backdrop, medical visit accompaniment services, as an emerging form of medical supportive service, have come into being and gradually begun to take shape as a new urban market (11, 12).

The core function of medical visit accompaniment services is to assist older patients throughout the entire care-seeking process, including registration, consultation, examination, medication collection, and follow-up visits, while also providing, when necessary, multi-level support such as information interpretation, psychological reassurance, and emotional companionship (13–15). In this way, such services can improve both the efficiency of diagnosis and treatment and the overall quality of the medical experience for older adults (16). However, a review of the existing literature indicates that China’s medical visit accompaniment service industry is still at an early stage of development. It is currently characterized by low willingness to use, inconsistent service standards, limited professionalization, unclear pricing mechanisms, and inadequate privacy protection, indicating that the overall service system remains immature (17–19).

Although medical visit accompaniment services have emerged in China as a relatively new market-oriented form of medical supportive service, the underlying demand for navigation, accompaniment, and care coordination among older adults has also been widely discussed in other aging societies. In Japan, studies on family caregivers and community-based long-term care have shown that older adults often require practical support in transportation, service coordination, and access to medical and long-term care resources (20, 21). In South Korea, the development of long-term care insurance has expanded formal care provision, but the separation between medical services and long-term care services continues to create challenges for continuity of care (22, 23). In Western Europe, research on integrated health and social care, as well as patient navigation, has emphasized the importance of helping older adults and their families overcome fragmented service systems and coordinate care across institutional boundaries (24–26). These international findings suggest that medical visit accompaniment services should not be understood merely as a local response to family caregiving constraints, but also as part of a broader global effort to improve healthcare accessibility, continuity of care, and age-friendly service delivery for older adults.

Nanjing was chosen as the sample city for this study because of its abundant medical resources and pronounced population aging. The city is also actively exploring the integration of smart healthcare and older adult care services. Therefore, Nanjing provides a representative and informative setting for this research (27, 28). Based on questionnaire data from 638 adult children of older adults in Nanjing, this study constructed a structural equation model to identify the key factors influencing willingness to use medical visit accompaniment services. This study enriches the theoretical framework of research on medical support services and provides empirical evidence for the transition of such services from a supply-oriented model to a demand-oriented one. The findings also offer practical implications for policy and service development.

## 2 Participants and methods

### 2.1 Participants

Adult children are often the primary decision-makers and financial supporters when older adults seek medical care. Their preferences therefore play an important role in shaping the market acceptance of medical visit accompaniment services. From March to May 2025, this study recruited 638 adult children of older adults from the 11 municipal districts of Nanjing. The study examined their actual needs for medical visit accompaniment services, their willingness to use such services, their service preferences, and the key factors influencing their behavioral decision-making. The inclusion criteria were as follows: (1) one or both parents were aged 60 years or above; (2) the respondent had accompanied their parent(s) to seek medical care within the past year; and (3) the respondent provided informed consent and participated voluntarily.

### 2.2 Methods

#### 2.2.1 Survey instrument

Data were collected using a structured questionnaire. The questionnaire was designed with reference to the results of preliminary focus group discussions with 10 members of the target population, through which the core dimensions of demand most valued by adult children in relation to medical visit accompaniment services were initially identified, including process assistance, medical interpretation, emotional support, and trust assurance. On this basis, in combination with the functional classification framework of medical visit accompaniment services reported in the relevant literature, and incorporating the Theory of Planned Behavior, a scale measuring willingness to use medical visit accompaniment services and a scale measuring service content needs were developed, thereby establishing the structural logic and variable design of the questionnaire (29, 30).

The questionnaire consisted of five parts: (1) Basic information, including sociodemographic variables such as gender, age, educational level, occupation, family structure, monthly income, and the frequency of parents’ medical visits; (2) Use of and perceptions of medical visit accompaniment services, including basic behavioral and cognitive variables such as whether the respondent had previous experience with medical visit accompaniment services, which service functions had been used, and the respondent’s attitudes toward such services; (3) A scale of factors influencing willingness to use medical visit accompaniment services, designed using a five-point Likert scale, with 15 observed variables measuring the latent constructs of attitude toward behavior, subjective norm, and perceived behavioral control; (4) A scale of service content needs for medical visit accompaniment services, including 15 observed variables measuring the latent constructs of basic support needs, emotional and dignity-related needs, and health management needs; (5) Future expectations regarding the use of medical visit accompaniment service platforms, including six observed variables: Frequency of Use, Health Considerations, Social Recognition, Work-Related Impact, Parents’ Attitudes, Adult Children’s Attitudes.

The questionnaire showed high internal consistency, with an overall Cronbach’s alpha coefficient of 0.972. The Kaiser–Meyer–Olkin (KMO) value was 0.968, indicating that the data were suitable for further factor analysis. However, because very high reliability values may also suggest potential overlap or redundancy among some items, the results were interpreted with caution. The questionnaire was developed through focus group discussions, reference to existing literature, and theoretical adaptation based on the Theory of Planned Behavior. Confirmatory factor analysis was further conducted to examine the structural validity of the measurement model.

#### 2.2.2 Survey procedure

To ensure that the sample was sufficiently representative of the target population while also balancing efficiency and cost, this study adopted a combination of stratified sampling, quota sampling, and snowball sampling. In the first stage, the 11 municipal districts of Nanjing were used as natural stratification units based on the city’s administrative divisions, and quota sampling was then conducted in each district according to population size. In the second stage, snowball sampling was employed: some respondents were initially selected according to the predetermined indicators to complete the questionnaire, and they were then asked to recommend other individuals who belonged to the target population of the study.

The questionnaire was distributed through both online and offline channels. Skip logic embedded in the Wenjuanxing survey platform was used to guide respondents to complete the questionnaire correctly, thereby ensuring the validity and reliability of the survey content.

A total of 58 questionnaires were distributed in the pilot survey, of which 56 were valid. In the formal survey, 659 questionnaires were collected through both online and offline channels. After excluding questionnaires with missing information, logical inconsistencies, repeated submissions, or highly patterned responses, 638 valid questionnaires were retained for the final analysis, yielding a valid questionnaire rate of 96.8% among returned questionnaires. This figure refers to the proportion of valid questionnaires among returned questionnaires rather than a conventional response rate calculated from all individuals initially approached. This relatively high valid questionnaire rate was mainly attributable to eligibility screening before questionnaire completion, skip-logic settings in the online questionnaire, and the exclusion of invalid questionnaires according to predefined quality-control criteria.

#### 2.2.3 Statistical analysis

An Excel database was established, and a structural equation model was constructed using Amos 28.0 to examine the mechanisms influencing willingness to use medical visit accompaniment services.

The model fit indices included the chi-square/degrees of freedom ratio (CMIN/DF), comparative fit index (CFI), and incremental fit index (IFI). In addition, the significance of the path coefficients for each variable was tested using *p* values, and the direct, indirect, and total effects of the independent variables were decomposed and analyzed to clarify the mechanisms and pathways through which each factor influenced willingness to use the services (31–33).

### 2.3 Ethics statement

This study employed an anonymous, non-interventional questionnaire survey and did not collect any personally identifiable information. Before completing the questionnaire, all participants were informed of the study purpose, the voluntary nature of participation, and their right to withdraw at any time. Completion of the questionnaire was regarded as informed consent. The study involved minimal risk and did not include any clinical intervention or collection of sensitive identifiable data. According to the regulations of the authors’ affiliated institution, formal ethical approval was not required for this type of anonymous, minimal-risk survey.

## 3 Model construction

### 3.1 Theoretical model

**Figure 1:**
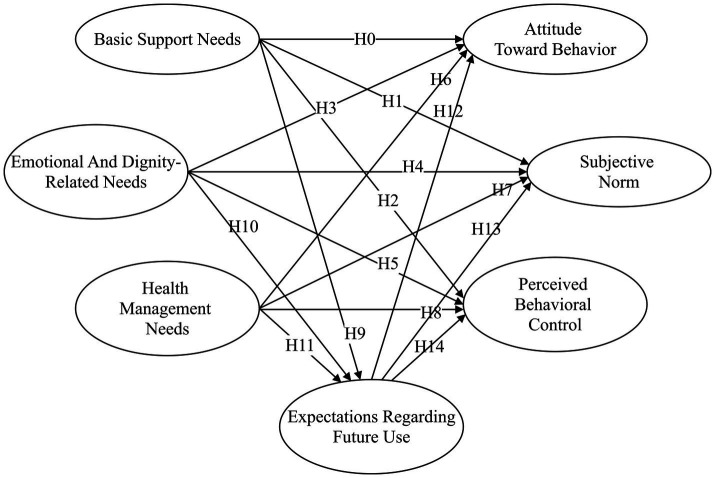
The theoretical structural model of the mechanisms influencing willingness to use medical visit accompaniment services.

This study integrated the Theory of Planned Behavior (TPB) (34–36) with the relevant literature (37–41) to construct a Structural Equation Modeling (SEM) framework, with the aim of examining the mechanisms influencing adult children’s willingness to use medical visit accompaniment services for older adults. In this study, it was hypothesized that three types of needs—basic support needs, emotional and dignity-related needs, and health management needs (12, 42, 43)—could not only directly influence adult children’s willingness to use medical visit accompaniment service platforms through attitude toward behavior, subjective norm, and perceived behavioral control, but could also indirectly affect such willingness by shaping their expectations regarding future use of medical visit accompaniment services (44–46). On this basis, a theoretical structural model of the mechanisms influencing willingness to use medical visit accompaniment services was developed. Figure 1 illustrates the hypothesized relationships among service needs, TPB-related cognitive variables, future expectations, and willingness to use medical visit accompaniment services.

### 3.2 Construction of the measurement index system

Details of the measurement index system are shown in Table 1.

**Table 1:** Construction of the measurement index system for medical visit accompaniment services.

Primary dimension	Latent variable	Observed variable
Willingness to use medical visit accompaniment services	Attitude toward behavior	Emergency assurance
		Emotional compensation
		Reliability and standardization
		Medical visit efficiency
		Medical visit experience
	Subjective norm	Family support
		Authoritative recommendation
		Physician recommendation
		Trend recognition
		Social influence
	Perceived behavioral control	Willingness to try new channels
		Budget consideration
		Problem-solving capacity
		Information acquisition
		Matching preference
Content needs for medical visit accompaniment services	Basic support needs	Basic assistance
		Emergency response
		First-aid competence
		Price transparency
		Privacy protection
	Emotional and dignity-related needs	Emotional support
		Respect for autonomy
		Dignity preservation
		Communication preferences
		Family feedback
	Health management needs	Medical record management
		Disease management
		Medication reminders
		Medical terminology interpretation
		Health Promotion
Future expectations regarding medical visit accompaniment services	Future expectations	Frequency of use
		Health considerations
		Social recognition
		Work-related impact
		Parents’ attitudes
		Adult children’s attitudes

### 3.3 Model estimation

#### 3.3.1 Model fit assessment

Confirmatory factor analysis (CFA) was conducted to evaluate the structural equation model of the mechanisms influencing willingness to use medical visit accompaniment services. The results showed that CMIN/DF = 3.953, RMSEA = 0.097, CFI = 0.827, IFI = 0.828, and TLI = 0.812. These values suggest that the model achieved an acceptable but not optimal level of fit. Although some fit indices were within commonly used acceptable ranges, others indicated room for improvement. Thus, the model should be regarded as providing an exploratory explanation of the relationships among the variables rather than a definitive confirmatory model.

#### 3.3.2 Results of hypothesis testing for the structural paths

As shown in Table 2, in the structural path analysis conducted in this study, the arrows indicate the hypothesized directions of association among variables, and the numerical values represent the path estimates. The estimated paths were statistically significant (*p* < 0.001), supporting the hypothesized associations among the variables.

**Table 2:** Results of the SEM path analysis of factors influencing willingness to use medical visit accompaniment service platforms (revised).

Structural path	Estimate	Standard error	Critical ratio	*p*
Attitude toward Behavior ← Basic Support Needs	1.793	0.299	12.594	< 0.001
Subjective Norm ← Basic Support Needs	1.784	0.307	12.629	< 0.001
Perceived Behavioral Control ← Basic Support Needs	1.795	0.295	12.348	< 0.001
Attitude toward Behavior ← Emotional and Dignity-Related Needs	−1.015	0.145	−13.106	< 0.001
Subjective Norm ← Emotional and Dignity-Related Needs	−0.980	0.148	−12.827	< 0.001
Perceived Behavioral Control ← Emotional and Dignity-Related Needs	−1.077	0.145	−13.389	< 0.001
Attitude toward Behavior ← Health Management Needs	−0.813	0.154	−10.521	< 0.001
Subjective Norm ← Health Management Needs	−0.844	0.160	−10.905	< 0.001
Perceived Behavioral Control ← Health Management Needs	−0.791	0.152	−10.04	< 0.001
Future Expectations ← Basic Support Needs	−0.765	0.103	−17.027	< 0.001
Future Expectations ← Emotional and Dignity-Related Needs	0.393	0.061	13.147	< 0.001
Future Expectations ← Health Management Needs	0.403	0.067	13.136	< 0.001
Attitude toward Behavior ← Future Expectations	1.388	0.131	9.711	< 0.001
Subjective Norm ← Future Expectations	1.370	0.135	9.638	< 0.001
Perceived Behavioral Control ← Future Expectations	1.436	0.130	9.774	< 0.001

**Figure 2:**
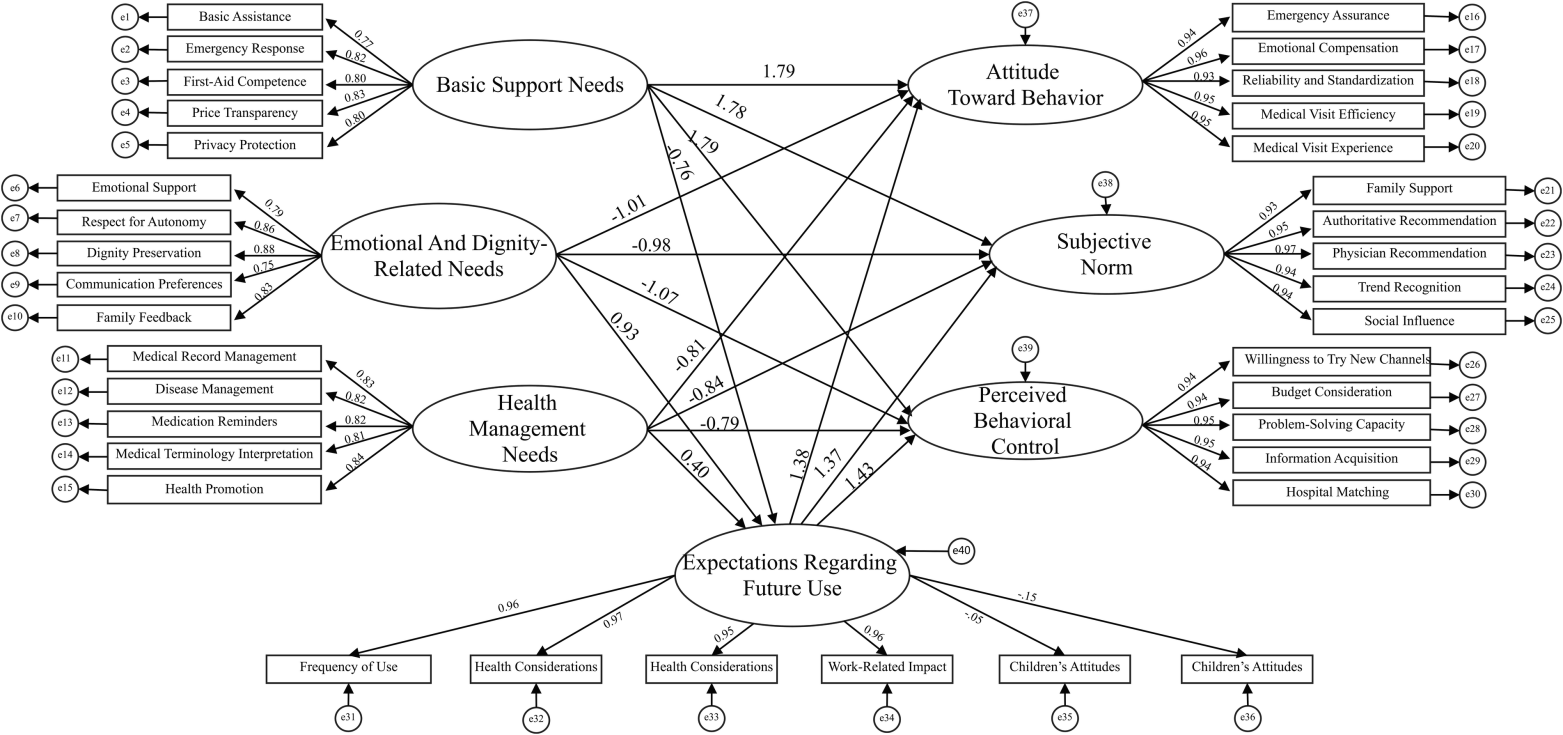
Structural equation model of the mechanisms influencing willingness to use medical visit accompaniment services.

Figure 2 presents the estimated SEM paths and shows the direction and estimated magnitude of the associations among the latent variables.

## 4 Results

### 4.1 Demographic characteristics of the study participants

As shown in Table 3, among the 638 respondents, 336 were male (52.66%) and 302 were female (47.34%). A total of 205 respondents (32.13%) were aged 30 years or below, 139 (21.79%) were aged 31–40 years, 154 (24.14%) were aged 41–50 years, and 140 (21.94%) were aged 51 years or above. In terms of educational attainment, the largest proportions had completed senior high school/technical secondary school education (31.97%) or junior college education (26.02%). Approximately 80% of the adult children of older adults had a monthly income above RMB 3,000. In addition, 67% of the respondents had siblings. More than 70% of the older adults were not accompanied at every medical visit, and 34.64% were accompanied by their adult children.

**Table 3:** Demographic characteristics of the study participants.

Characteristic	Number of participants	Percentage (%)
Gender		
Male	336	52.66
Female	302	47.34
Age		
≤30 years	205	32.13
31–40 years	139	21.79
41–50 years	154	24.14
≥51 years	140	21.94
Educational level		
Junior high school or below	59	9.25
Senior high school/technical secondary school	204	31.97
Junior college	166	26.02
Bachelor’s degree	155	24.29
Master’s degree or above	54	8.46
Marital status		
Unmarried	205	32.13
Married	352	55.17
Divorced	54	8.46
Widowed	27	4.23
Monthly income		
≤ RMB 3,000	126	19.75
RMB 3,001–5,001	184	28.84
RMB 5,001–10,000	170	26.65
RMB 10,001–30,000	102	15.99
≥ RMB 30,001	56	8.78
Siblings		
Yes	433	67.87
No	205	32.13
Whether older adults were accompanied during medical visits		
Accompanied at every visit	156	24.45
Often accompanied	219	34.33
Occasionally accompanied	178	27.90
Rarely accompanied	85	13.32
Primary accompanying person		
Spouse	151	23.67
Children	221	34.64
Other relatives	125	19.59
Caregivers/medical visit accompaniment providers	79	12.38
Friends/neighbors	62	9.27

### 4.2 Analysis of the model results

To further examine the specific pathways through which different types of demand for medical visit accompaniment services influenced willingness to use such services, this study conducted an effect decomposition analysis of the structural equation model. Specifically, the direct path coefficient was defined as the path coefficient from the independent variable to the dependent variable; the indirect path coefficient was defined as the product of the path coefficient from the independent variable to the mediating variable and the direct path coefficient of the mediating variable; and the total path coefficient was defined as the sum of the direct and indirect path coefficients. The results are presented in Table 4.

**Table 4:** Effect decomposition based on unstandardized SEM path estimates.

Variable	Direct effect path	Direct effect coefficient	Indirect effect path	Indirect effect coefficient	Total effect
Basic support needs	Basic Support Needs → Attitude toward Behavior	1.793	Basic Support Needs → Future Expectations → Attitude toward Behavior	−1.142	0.651
	Basic Support Needs → Subjective Norm	1.784	Basic Support Needs → Future Expectations → Subjective Norm	−1.365	0.419
	Basic Support Needs → Perceived Behavioral Control	1.795	Basic Support Needs → Future Expectations → Perceived Behavioral Control	−1.373	0.422
	**Basic Support Needs → Willingness to Use**	**5.372**	**Basic Support Needs → Future Expectations → Willingness to Use**	**−3.880**	**1.492**
Emotional and dignity-related needs	Emotional and Dignity-Related Needs → Attitude toward Behavior	−1.015	Emotional and Dignity-Related Needs → Future Expectations → Attitude toward Behavior	−0.399	−1.414
	Emotional and Dignity-Related Needs → Subjective Norm	−0.980	Emotional and Dignity-Related Needs → Future Expectations → Subjective Norm	−0.385	−1.365
	Emotional and Dignity-Related Needs → Perceived Behavioral Control	−1.077	Emotional and Dignity-Related Needs → Future Expectations → Perceived Behavioral Control	−0.423	−1.500
	**Emotional and Dignity-Related Needs → Willingness to Use**	**−3.072**	**Emotional and Dignity-Related Needs → Future Expectations → Willingness to Use**	**−1.207**	**−4.279**
Health management needs	Health Management Needs → Attitude toward Behavior	−0.813	Health Management Needs → Future Expectations → Attitude toward Behavior	−0.328	−1.141
	Health Management Needs → Subjective Norm	−0.844	Health Management Needs → Future Expectations → Subjective Norm	−0.340	−1.184
	Health Management Needs → Perceived Behavioral Control	−0.791	Health Management Needs → Future Expectations → Perceived Behavioral Control	−0.319	−1.110
	**Health Management Needs → Willingness to Use**	**−2.448**	**Health Management Needs → Future Expectations → Willingness to Use**	**−0.987**	**−3.435**

Bold values indicate the total effect of each factor on willingness to use medical visit accompaniment services, calculated as the sum of the direct and indirect effects based on unstandardized SEM path estimates.

It should be noted that the coefficients reported in the effect decomposition are based on model path estimates and cumulative direct and indirect effects. Therefore, relatively large values should not be interpreted as standardized effect sizes, but rather as indicators of the relative direction and magnitude of associations within the specified model.

Basic support needs, emotional and dignity-related needs, and health management needs all exerted effects through both direct pathways and the mediating variable of future expectations. Their effects showed significant differences and substantial explanatory value.

1. Basic support needs had a positive effect on willingness to use medical visit accompaniment services (total path coefficient = 1.492). Along the direct path, the path coefficient from this variable to willingness to use was 5.372, while an indirect effect of −3.880 was observed through the mediating variable of future expectations, resulting in a statistically significant overall positive effect. This indicates that adult children’s recognition of basic service functions, such as process assistance, registration support, and medical information organization, is the primary driver of increased willingness to use medical visit accompaniment services (11, 16). Although anxiety regarding future care expectations may attenuate part of this positive effect, the overall influence remains strongly positive.
2. Emotional and dignity-related needs showed a marked negative total effect on willingness to use medical visit accompaniment services (−4.279), consisting of a direct effect (−3.072) and an indirect effect (−1.207). This finding reflects a phenomenon of unmet emotional expectations. Although users have relatively high expectations regarding humanistic companionship, respect for dignity, and other emotional dimensions of medical visit accompaniment services, the current service market has not yet been able to adequately meet such emotional needs, thereby producing a negative behavioral response (47). This psychological discrepancy mechanism suggests that when emotional needs are not effectively fulfilled, individuals may develop negative psychological reactions because of the gap between expectation and reality, which in turn reduces their willingness to accept the service. However, this negative association should be interpreted cautiously. In addition to unmet emotional expectations, it may also be related to measurement overlap among service need dimensions, respondents’ different understandings of emotionally oriented services, or limitations in the current model specification.
3. Health management needs also exerted a negative effect, with a total effect of −3.435. This variable showed negative coefficients across the pathways of attitude toward behavior, subjective norm, and perceived behavioral control, and also generated an additional negative effect through the pathway of future expectations. These results suggest that the current supply structure of medical visit accompaniment services has not yet been effectively aligned with health management-oriented needs. In particular, deficiencies in service functions such as chronic disease follow-up support and health information consultation have weakened users’ expected evaluation of the potential benefits of using such services (48, 49). Similarly, the negative effect of health management needs may reflect not only insufficient supply of health-management-oriented services, but also possible measurement limitations, high user expectations for professional services, or the early developmental stage of the medical visit accompaniment service market.

## 5 Discussion

### 5.1 Principal findings

#### 5.1.1 The market positioning of medical visit accompaniment services as a process-oriented healthcare service remains unclear

The model results showed that willingness to use medical visit accompaniment services was significantly influenced by factors such as attitude toward behavior, subjective norm, and perceived behavioral control, indicating that this type of service does not essentially belong to the category of conventional medical care, but is more appropriately regarded as a form of medical support service. In China, such services have not yet developed clear pricing mechanisms, entry standards, or regulatory boundaries (50, 51), resulting in an ambiguous market position and substantial fluctuations in users’ trust and acceptance (52). These findings indicate that unclear service boundaries, billable elements, and regulatory standards may increase information asymmetry and weaken users’ trust in medical visit accompaniment services.

#### 5.1.2 The dilemma of low market returns for emotionally oriented health services reflects a disconnect between service value and payment mechanisms

Medical visit accompaniment services can provide emotional support and assistance to older patients, helping to alleviate negative emotions such as tension, anxiety, and fear, while enhancing their psychological resilience and self-healing capacity (53, 54). However, the model results revealed a pronounced negative effect, suggesting that such needs are not currently being effectively addressed. This stems from the fact that emotionally oriented services often generate high marginal benefits and high user satisfaction, yet remain trapped in a market dilemma of low returns and difficult pricing because they are not easily standardized (47, 55). Under market mechanisms, these services lack sufficient supply-side incentives, and under the medical insurance system, they are also difficult to incorporate into formal pricing schedules, leaving them in an institutional gray zone. Therefore, establishing incentive and payment mechanisms targeted at emotional care needs will be a critical issue that must be addressed in the future institutionalization of medical visit accompaniment services (56, 57).

#### 5.1.3 The positive externalities of medical visit accompaniment services need to be internalized through socialized mechanisms and tiered service strategies

Users’ willingness to use medical visit accompaniment services is influenced not only by the service content itself, but also closely related to broader social expectations. This suggests that medical visit accompaniment services possess certain positive externalities, as they can improve the efficiency of older adults’ medical visits, reduce family caregiving burdens, and minimize the waste of medical resources, thereby generating systemic benefits (58, 59). At present, however, such services still rely primarily on out-of-pocket choices made by individual families and lack broader social support mechanisms.

### 5.2 Comparison with international research

The findings of this study are broadly consistent with international research on patient navigation, integrated care, and long-term care support for older adults. Studies on patient navigation have shown that navigation services can help patients and families overcome barriers in complex health systems, especially when care pathways involve multiple providers, fragmented information, and repeated transitions between institutions. The strong positive effect of basic support needs found in this study echoes this international evidence, indicating that process assistance and service coordination are central components of supportive care for older adults (60, 61).

Evidence from Japan, South Korea, and Western Europe further highlights the importance of viewing medical visit accompaniment services within a broader aging-service system (62). In Japan, family caregivers often play a crucial role in helping older adults access healthcare and long-term care services, particularly when transportation, service coordination, and information communication are required (21). In South Korea, long-term care insurance has expanded formal service provision, but the separation between medical care and long-term care continues to create challenges for integrated support (63). In Western Europe, research on integrated health and social care has emphasized that older adults’ needs often extend beyond clinical treatment and require coordinated support across medical, social, and community-based sectors (64). Compared with these international models, medical visit accompaniment services in China are still at an early stage of professionalization. Their current strength lies mainly in basic procedural assistance, whereas their capacity to provide emotional support, dignity-oriented care, and health management remains insufficient (65, 66).

### 5.3 Implications for public health policy and practice

Medical visit accompaniment services should be incorporated into the broader construction of age-friendly healthcare and older adult care systems. Given that basic support needs were the strongest positive driver of willingness to use, policy and service planning should give priority to standardized process-oriented support for registration, hospital navigation, examination coordination, and medical information organization. Emotional support, dignity protection, chronic disease follow-up, and health information interpretation should be developed gradually as advanced service modules, with clear professional boundaries, service standards, and personnel training to avoid unrealistic expectations or role ambiguity.

Beyond service-level optimization, the development of medical visit accompaniment services requires a collaborative mechanism based on institutional incentives and multi-stakeholder participation, so as to recognize and internalize the positive externalities of these services. Community organizations, volunteer groups, and enterprises may be encouraged to provide certain basic accompaniment services through public welfare initiatives, thereby improving social accessibility (67, 68). Meanwhile, a transparent and stratified pricing system should be fostered within the market, allowing groups with different income levels to obtain services according to differentiated needs and avoiding the imbalance between inadequate basic services and excessive high-end services. Local governments may also strengthen policy support for specific populations by providing caregiving subsidies or integrating medical visit accompaniment services into family care programs, thereby improving service equity, social recognition, and the flexibility of public support systems (52).

### 5.4 Limitations

This study has several limitations. Although stratified and quota sampling were used to improve geographical coverage across the 11 municipal districts of Nanjing, the use of snowball sampling may have introduced non-probability sampling bias. Respondents recruited through social networks may share similar caregiving experiences or attitudes toward medical visit accompaniment services, which may limit the generalizability of the findings. Therefore, the sample should be interpreted as a city-based empirical sample with improved geographical coverage rather than a fully representative probability sample of all adult children of older adults in Nanjing or China. In addition, this study was based on a single-city, cross-sectional survey; therefore, future multi-city and longitudinal studies are needed to further validate the robustness and generalizability of the findings.

## 6 Conclusion

This study constructed a structural equation model to examine the mechanisms influencing adult children’s willingness to use medical visit accompaniment services for older adults in Nanjing. The results show that basic support needs are the strongest positive driver of willingness to use such services, whereas emotional and dignity-related needs and health management needs show negative effects because of the current mismatch between user expectations and service supply capacity. These findings suggest that the development of medical visit accompaniment services should begin with standardized basic procedural support and gradually expand toward emotional support, dignity-oriented care, and health management functions. From a public health perspective, well-regulated medical visit accompaniment services may help improve healthcare accessibility for older adults, reduce family caregiving pressure, and promote the construction of age-friendly healthcare systems.

## Data Availability

The raw data supporting the conclusions of this article will be made available by the authors, without undue reservation.
